# Loss of Non-Apoptotic Role of Caspase-3 in the PINK1 Mouse Model of Parkinson’s Disease

**DOI:** 10.3390/ijms20143407

**Published:** 2019-07-11

**Authors:** Paola Imbriani, Annalisa Tassone, Maria Meringolo, Giulia Ponterio, Graziella Madeo, Antonio Pisani, Paola Bonsi, Giuseppina Martella

**Affiliations:** 1 Laboratory of Neurophysiology and Plasticity, IRCCS Fondazione Santa Lucia, 00143 Rome, Italy; 2 Department of Systems Medicine, University of Rome “Tor Vergata”, 00133 Rome, Italy

**Keywords:** Parkinson’s disease, PINK1, caspase-3, striatum, synaptic plasticity, long-term depression

## Abstract

Caspases are a family of conserved cysteine proteases that play key roles in multiple cellular processes, including programmed cell death and inflammation. Recent evidence shows that caspases are also involved in crucial non-apoptotic functions, such as dendrite development, axon pruning, and synaptic plasticity mechanisms underlying learning and memory processes. The activated form of caspase-3, which is known to trigger widespread damage and degeneration, can also modulate synaptic function in the adult brain. Thus, in the present study, we tested the hypothesis that caspase-3 modulates synaptic plasticity at corticostriatal synapses in the phosphatase and tensin homolog (PTEN) induced kinase 1 (PINK1) mouse model of Parkinson’s disease (PD). Loss of PINK1 has been previously associated with an impairment of corticostriatal long-term depression (LTD), rescued by amphetamine-induced dopamine release. Here, we show that caspase-3 activity, measured after LTD induction, is significantly decreased in the PINK1 knockout model compared with wild-type mice. Accordingly, pretreatment of striatal slices with the caspase-3 activator α-(Trichloromethyl)-4-pyridineethanol (PETCM) rescues a physiological LTD in PINK1 knockout mice. Furthermore, the inhibition of caspase-3 prevents the amphetamine-induced rescue of LTD in the same model. Our data support a hormesis-based double role of caspase-3; when massively activated, it induces apoptosis, while at lower level of activation, it modulates physiological phenomena, like the expression of corticostriatal LTD. Exploring the non-apoptotic activation of caspase-3 may contribute to clarify the mechanisms involved in synaptic failure in PD, as well as in view of new potential pharmacological targets.

## 1. Introduction

Caspases are a highly conserved family of cysteine proteases playing a central role in the execution phase of apoptotic cell death [1,2]. Caspases are synthesized as catalytically inactive proenzymes and can be categorized in initiators and effectors. Initiator caspases (caspases-8, -9, -10, and -2) require autocatalytic cleavage for maturation. Effector caspases (caspases-3, -6, and -7) are activated by initiator caspases through a proteolytic cleavage and are responsible for degradation of structural proteins, signaling molecules, and DNA repair enzymes [3]. Active caspases have also been detected in non-apoptotic cells, including neurons, where they have been found to be active in dendrites, synapses, and growth cones [4,5,6,7], not involving death-related processes [8]. A member of this family, caspase-3, classically known as a key mediator of neuronal apoptosis, also exerts non-apoptotic functions [9,10]. In immature central nervous system, caspases-3 modulates structural functions, including developmental pruning of axons, dendrites, and synaptic connections, and is involved in axon degeneration induced by trophic factor withdrawal [11,12,13]. Mice lacking caspase-3 displays severe anomalies in the cerebral cortex and forebrain, owing to defects in nervous system development [14]. In the healthy adult brain, caspase-3 activity seems essential for physiological synaptic function and neurogenesis [15]. Both in vivo and in vitro findings support the involvement of caspase-3 in the molecular mechanisms underlying learning and memory processes [10,16]. Several caspase-3 substrates are key determinants of the molecular machinery of synaptic plasticity, including α-amino-3-hydroxy-5-methyl-4-isoxazolepropionic acid (AMPA) receptor subunit GluR1 [8,17,18,19]. Electrophysiological experiments on the terrestrial snail (Helix lucorum) showed that blockade of caspase-3 activity, obtained using the cell-permeable inhibitor Z-Devd-fmk, prevented the development of the long-term synaptic sensitization, indicating that caspase-3 is essential for long-term plasticity in invertebrate neurons [20]. A further recent electrophysiology study performed on rat hippocampal slices demonstrated that caspase-9-caspase-3 cascade, activated by pro-apoptotic molecules released from mitochondria, is required for the internalization of glutamatergic AMPA receptors from synapses and for the induction of long-term depression (LTD) [10]. Moreover, pretreatment of hippocampal slices with caspase inhibitors Z-Devd-fmk and Lehd-fmk prevents the induction of LTD at Schaffer collateral-CA1 synapses [10]. To our knowledge, the studies available so far have focused exclusively on the role of caspase-3 on hippocampal synaptic plasticity mechanisms. Here, we investigated whether caspase-3 plays a role in modulating synaptic plasticity expression at corticostriatal synapses in physiological and pathological conditions. To this aim, we used a well-characterized mouse model of autosomal recessive early-onset Parkinson’s disease (PD) [21], the phosphatase and tensin homolog (PTEN) induced kinase 1 (PINK1) knockout mouse model [22]. As previously demonstrated, homozygous PINK1 knockout (PINK1^−/−^) mice show an impaired expression of corticostriatal LTD [22], whereas LTD is preserved in heterozygous PINK1 (PINK1^+/−^) mice [23]. Thus, we explored whether caspase-3 is involved in the synaptic plasticity machinery underlying the expression of corticostriatal LTD in the PINK1 mouse model.

## 2. Results

### 2.1. Normal Intrinsic Membrane Properties of Striatal Medium Spiny Neurons (MSNs) after Pharmacological Modulation of Caspase-3

**Figure 1:**
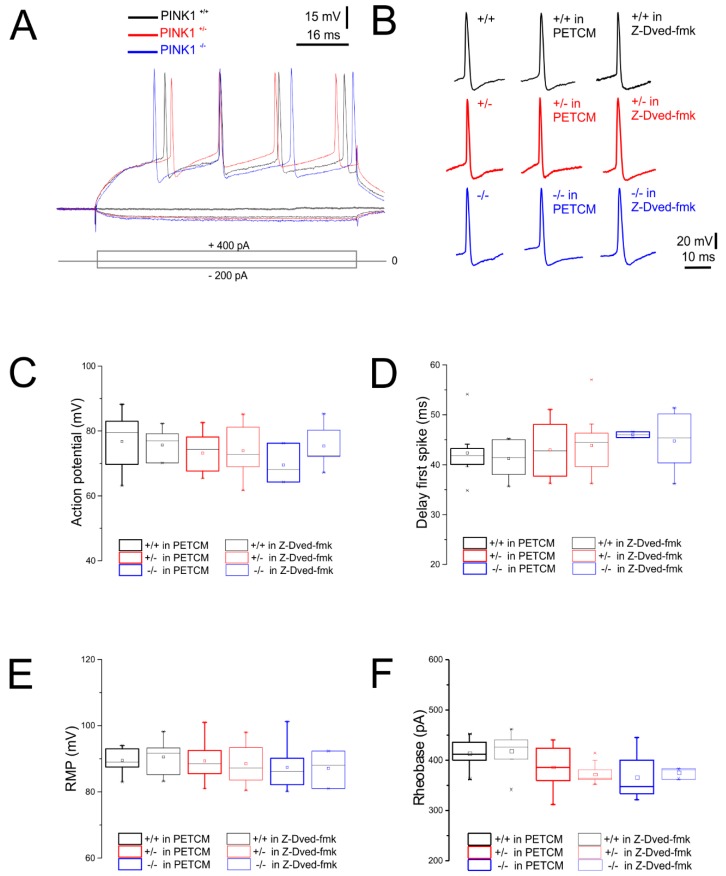
Intrinsic membrane properties of medium spiny neurons (MSNs) in phosphatase and tensin homolog (PTEN) induced kinase 1 (PINK1) mice after pretreatment with caspase-3 inhibitor and caspase-3 activator. (**A**) Superimposed traces showing voltage responses to current steps in both hyperpolarizing (−200 pA, 80 ms) and depolarizing (+400 pA, 80 ms) direction from PINK1^+/+^ (black trace), PINK1^+/−^ (red trace), and PINK1^−/−^ (blue trace) MSNs. No significant differences were observed between the three genotypes. (**B**) Representative single action potentials recorded from MSNs both in basal condition and after treatment with the caspase-3 inhibitor Z-Devd-fmk (5 µM) or with the caspase-3 activator α-(Trichloromethyl)-4-pyridineethanol (PETCM 30 µM) in all PINK1 genotypes. We did not observe significant differences between groups, in terms of upward spike, firing threshold, and half amplitude duration. (**C**–**F**) Whisker plots showing (**C**) action potential amplitude (PINK1^+/+^: 77.00 ± 3.8 mV; PINK1^+/−^: 78.4 ± 3.6 mV; PINK1^−/−^: 79.4 ± 4.02 mV; *n* = 9 for each group; one-way analysis of variance (ANOVA) *p* > 0.05), (**D**) delay to first spike (PINK1^+/+^: 40.65 ± 1.99 ms; PINK1^+/−^: 42.13 ± 1.6 ms; PINK1^−/−^: 44.23 ± 2.01 ms; *n* = 9 for each group; one-way ANOVA *p* > 0.05), (**E**) resting membrane potential (RMP) (PINK1^+/+^: −85 ± 3.8 mV; PINK1^+/−^: −87.21 ± 3.6 mV; PINK1^−/−^: −79.4 ± 4.02 mV; *n* = 9 for each group; one-way ANOVA; RMP Z-Dved-fmk on PINK1^+/+^: −90.53 ± 1.59 mV; Z-Dved-fmk on PINK1^+/−^: −88.51 ±1.60 mV; Z-Dved-fmk on PINK1^−/−^: −87.10 ± 3.1 mV; *n* = 9 for each group; two-way ANOVA *p* > 0.05), and (**F**) Rheobase (PINK1^+/+^: 4201 ± 12.1 ms; PINK1^+/−^: 400 ± 18.1 ms; PINK1^−/−^: 380 ± 18 ms; *n* = 9 for each group; one-way ANOVA *p* > 0.05) values, without significant differences between genotypes, regardless of treatment with caspase-3 inhibitor/activator.

First, we explored whether caspase-3 modulation perturbs the intrinsic membrane properties of striatal MSNs (Table 1). MSNs’ membrane properties were analyzed from corticostriatal slices obtained from three different PINK1 genotypes, namely PINK1^+/+^, PINK1^+/−^, and PINK1^−/−^, as previously described [22,24,25,26]. Specifically, we measured evoked firing activity (Figure 1A,B), action potential amplitude (Figure 1C), delay to spike threshold (Figure 1D), resting membrane potential (RMP) (Figure 1E), and rheobase (Figure 1F). 

### 2.2. Unaltered Glutamatergic Short-Term Synaptic Plasticity after Pharmacological Modulation of Caspase-3

**Figure 2:**
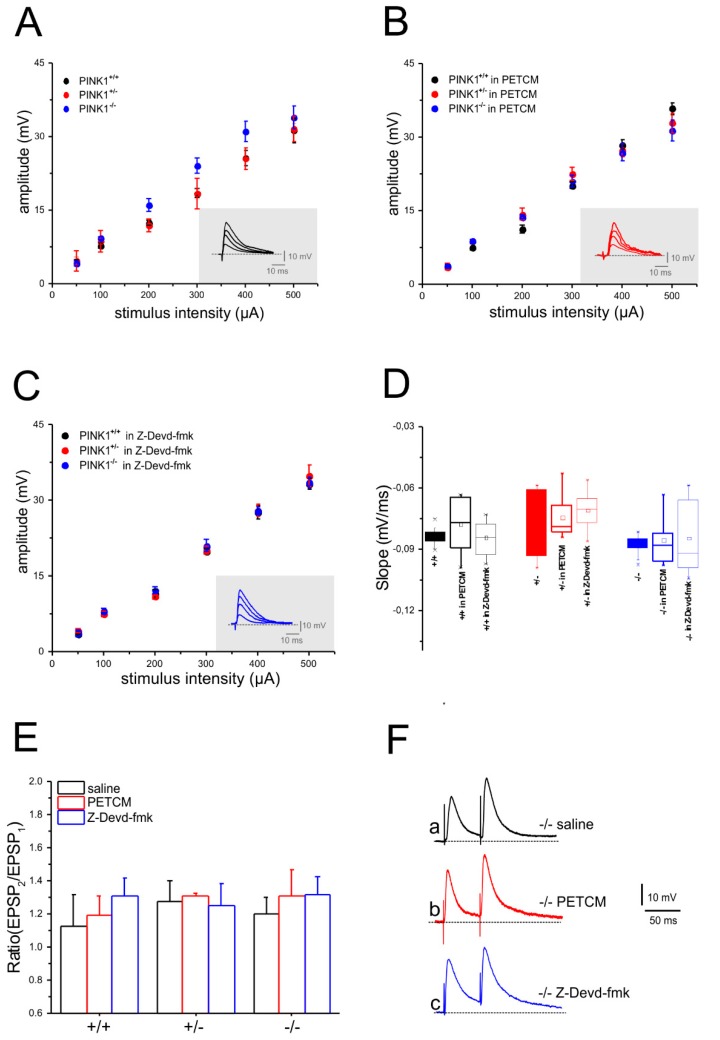
Pretreatment with caspase-3 inhibitor and activator does not affect evoked synaptic responses in PINK1 MSNs. Stimulation of corticostriatal fibers in the presence of Picrotoxin (PTX, 50 µM) produced glutamatergic excitatory postsynaptic potentials (EPSPs). Input–output (I/O) curves were built by measuring the amplitude of EPSPs evoked by increasing intensities of stimulation. (**A**) The I/O relationships were not significantly different between the three PINK1 genotypes. The inset shows representative superimposed traces of a single recording. (**B**) Treatment with PETCM (30 µM) did not induce significant differences neither among the three genotypes nor as compared with non-treated MSNs. (**C**) Similarly, treatment with Z-Devd-fmk (5 µM) did not affect the I/O curves in any experimental condition. (**D**) Plots showing EPSP slope values recorded from PINK1^+/+^ (black), PINK1^+/−^ (red), and PINK1^−/−^ (blue) MSNs treated with vehicle, PETCM, or Z-Devd-fmk. No significant differences were observed among groups (50 µA stimulus: PINK1^+/+^: 3.84 ± 0.27 mV/ms, *n* = 11; PINK1^+/−^: 3.5 ± 0.66 mV/ms, *n* = 10; PINK1^−/−^: 3.6 ± 0.3 mV/ms, *n* = 10; PINK1^+/+^ in Z-Devd-fmk: 4.21 ± 0.3 mV/ms, *n* = 10; PINK1^+/−^ in Z-Devd-fmk: 4.8 ± 0.61 mV/ms, *n* = 10; PINK1^−/−^ in Z-Devd-fmk: 4.01 ± 0.72 mV/ms, *n* = 8; PINK1^+/+^ in PETCM: 4.4 ± 0.6 mV/ms, *n* = 8; PINK1^+/−^ in PETCM: 4.78 ± 1.02 mV/ms, *n* = 7; PINK1^−/−^ in PETCM: 4.21 ± 0.53 mV/ms, *n* = 8; two-way ANOVA *p* > 0.05). (**E**,**F**) Paired-pulse facilitation (50 ms ISI) was similar in all PINK1 genotypes, both in saline solution and after pharmacological treatment. (**E**) The plot shows paired-pulse ratio (PPR) values, defined as EPSP2/EPSP1 ratio (PPR: PINK1^+/+^: 1.2 ± 0.16, *n* = 6; PINK1^+/−^: 1.23 ± 0.14, *n* = 6; PINK1^−/−^: 1.28 ± 0.12, *n* = 6; PINK1^+/+^ in Z-Devd-fmk: 1.28 ± 0.12, *n* = 9; PINK1^+/+^ in PETCM: 1.2 ± 0.10, *n* = 9; PINK1^+/−^ in Z-Devd-fmk: 1.32 ± 0.02, *n* = 9; PINK1^+/−^ in PETCM: 1.30 ± 0.16, *n* = 9; two-way ANOVA *p* > 0.05). (**F**) Representative paired recordings of EPSPs from PINK1^−/−^ MSNs after treatment with saline solution (a, black traces), PETCM (b, red traces), and Z-Devd-fmk (c, blue traces) (PPR: PINK1^−/−^ in saline: 1.32 ± 0.10, *n* = 9; PINK1^−/−^ in Z-Devd-fmk: 1.25 ± 0.14, *n* = 9; PINK1^−/−^ in PETCM: 1.32 ± 0.10, *n* = 9; one-way ANOVA *p* > 0.05).

Excitatory postsynaptic potentials (EPSPs) were evoked by 0.1 Hz stimulation of corticostriatal fibers (Figure 2A). Slice perfusion with either PETCM (Figure 2B,D) or Z-Devd-fmk (Figure 2C,D) did not modify amplitude and slope of the EPSPs recorded from MSNs of all genotypes (Figure 2B–D). The input–output relationship was obtained by increasing stimulus intensity and by measuring the peak amplitude and slope of the evoked EPSP. No significant differences among groups were observed (Figure 2A–D; *n* ≥ 6 for each condition, two-way ANOVA *p* > 0.05). Glutamatergic short-term synaptic plasticity was assessed with a paired-pulse protocol, consisting of paired stimulation (50 ms interstimulus interval, ISI) delivered at 0.1 Hz in picrotoxin (PTX, 50 µM). Paired stimulation produced a similar response in all genotypes [22,23,27]. A comparable facilitation of synaptic transmission (PPF) was induced in PINK1^−/−^ corticostriatal slices (Figure 2E) after incubation in saline solution (Figure 2Fa), PETCM (Figure 2Fb), or Z-Devd-fmk (Figure 2Fc). Similarly, PPR measured in PINK1^+/+^ and PINK1^+/−^ MSNs in the different experimental conditions did not show any significant differences (Figure 2E). 

### 2.3. Corticostriatal LTD Expression Requires Caspase-3 Activation

We previously reported the loss of both corticostriatal LTD and LTP in homozygous PINK1^−/−^ mice [2] and sparing of LTD in heterozygous PINK1^+/−^ mice [23]. Considering previous studies investigating the role of caspase-3 in the expression of hippocampal LTD [10], we aimed at assessing whether modulation of caspase-3 could influence the expression of corticostriatal LTD in the PINK1 mouse model. 

**Figure 3:**
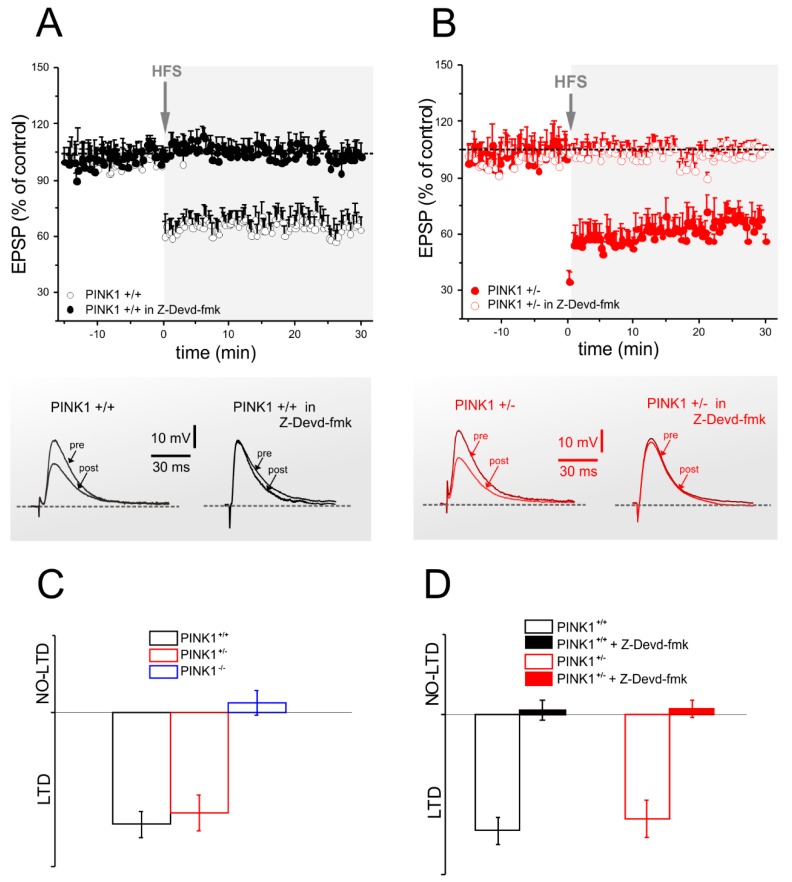
Long-term depression (LTD) in PINK1^+/+^ and PINK1^+/−^ mice is suppressed by caspase-3 inhibition. (**A**,**B**) Top. Time-course of LTD in PINK1^+/+^ and PINK1^+/−^ mice. Stimulus intensity was raised to reach threshold level for high-frequency stimulation (HFS). The amplitude of EPSPs was plotted over-time as percentage of the pre-HFS control EPSP. (**A**) HFS of corticostriatal glutamatergic afferents elicited a robust LTD in MSNs recorded from PINK1^+/+^ mice (black dots), but not after pretreatment with the caspase-3 inhibitor Z-Dved-fmk (5 µM) (white dots) (PINK1^+/+^ in Z-Devd-fmk: 98.25% ± 2.12% of control, *n* = 8; Student’s *t*-test *p* > 0.05; PINK1^+/+^ in saline: 63.70% ± 3.09% of control, *n* = 6; Student’s *t*-test *p* < 0.05). (**B**) Similarly, Z-Dved-fmk treatment suppressed LTD expression in PINK1^+/−^ mice (PINK1^+/−^ in Z-Devd-fmk: 105.69% ± 3.0% of control, *n* = 8, Student’s *t*-test *p* > 0.05; PINK1^+/−^ in saline: 61.44% ± 1.28% of control, *n* = 10; Student’s *t*-test *p* < 0.05). Each data point represents the mean ± SEM of eight independent observations for each group. Bottom. Sample traces of representative EPSPs recorded before (pre) and 30 min after (post) HFS in PINK1^+/+^ and PINK1^+/−^ mice, both in control condition and after treatment with Z-Dved-fmk. (**C**) Plot summarizing corticostriatal LTD expression in PINK1 mice. (**D**) The plot summarizes the loss of corticostriatal LTD after Z-Dved-fmk treatment in PINK1^+/+^ and PINK1^+/−^ genotypes.

**Figure 4:**
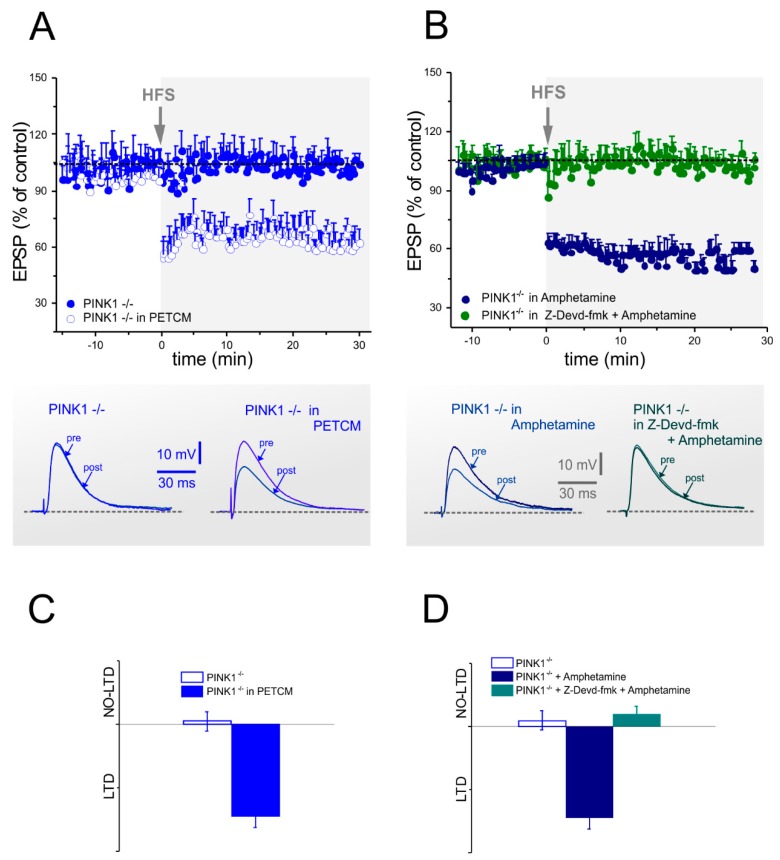
Long-term depression impairment in PINK1^−/−^ MSNs is rescued by caspase-3 activation. (**A**,**B**) Top. Time-course of LTD in PINK1^−/−^ mice. (**A**) HFS-protocol did not elicit LTD in MSNs recorded from PINK1^−/−^ mice (blue dots), but induced the expression of physiological LTD after pretreatment with the caspase-3 activator PETCM (30 µM) (empty dots) (PINK1^−/−^ in PETCM: 61.63% ± 1.40% of control, *n* = 8; Student’s *t*-test *p* < 0.05; PINK1^−/−^ in saline: 98.55% ± 2.7.% of control, *n* = 6; Student’s *t*-test *p* > 0.05). (**B**) Treatment whit amphetamine (100 µM) was also able to rescue LTD in PINK1^−/−^ mice (dark blue dots). However, combined treatment with amphetamine plus Z-Dved-fmk failed to rescue LTD in the same PINK1 genotype (green dots) (PINK1^−/−^ in amphetamine: 60.15% ± 3.67% of control, *n* = 6; Student’s *t*-test *p* < 0.05; PINK1^−/−^ in Z-Devd-fmk + amphetamine: 101% ± 3.08% of control, *n* = 6; Student’s *t*-test *p* > 0.05). Bottom. Sample traces of representative EPSPs recorded before (pre) and 30 min after (post) HFS in PINK1^−/−^ mice, in all the described experimental conditions. (**C**,**D**) The plots summarize the rescue of corticostriatal LTD after either PETCM or amphetamine treatment and the loss of LTD after combined treatment with amphetamine plus Z-Dved-fmk in PINK1^−/−^ mice.

In a first set of experiments, we assessed whether the inhibition of caspase-3 by Z-Devd-fmk could disrupt the physiological LTD observed both in PINK1^+/+^ and PINK1^+/−^ mice (Figure 3A–C). Incubation in Z-Devd-fmk (5 µM/1 h) of PINK1^+/+^ (Figure 3A,D) and PINK1^+/−^ (Figure 3B,D) corticostriatal slices prevented LTD expression. In light of these results, supporting caspase-3 requirement for corticostriatal LTD expression, we tested whether the activation of caspase-3 could be sufficient to rescue LTD defect in PINK1^−/−^ neurons. Therefore, PINK1^−/−^ corticostriatal slices were treated with the caspase-3 activator PETCM. Indeed, in this condition, we obtained a complete rescue of LTD expression in PINK1^−/−^ MSNs (Figure 4A,C). Our previous results showed that amphetamine was able to rescue LTD in PINK1^−/−^ corticostriatal slices. Thus, we performed further experiments in the presence of the caspase-3 inhibitor Z-Devd-fmk plus amphetamine, to verify if the activation of caspase-3 is involved in the amphetamine-mediated rescue of LTD. These experiments showed that when caspase-3 activation was blocked, amphetamine was no longer able to rescue LTD in PINK1^−/−^ MSNs (Figure 4B,D). 

### 2.4. Caspase-3 Activity is Lower in PINK1^−/−^ Mice after High Frequency Stimulation

In addition to electrophysiological experiments, we performed a colorimetric assay in order to measure caspase-3 activity in all PINK1 genotypes. First, we analyzed striatal slices from wild type mice, incubated with high doses of PETCM (100 µM, 1 h, 37 °C), and compared them with naïve samples (untreated slices). As expected, PETCM was able to induce a drastic increase in caspase-3 activity (Figure S1; naïve absorbance = 0.0285 ± 0.0077, *n* = 3; PETCM absorbance = 0.14550 ± 0.01768; *n* = 3; Student’s *t*-test *p* < 0.05), supporting the reliability of the assay. The levels of caspase-3 activation measured after PETCM treatment were in accordance with caspase-3 assay performed on cell lysates and suggestive of apoptosis [24].

**Figure 5:**
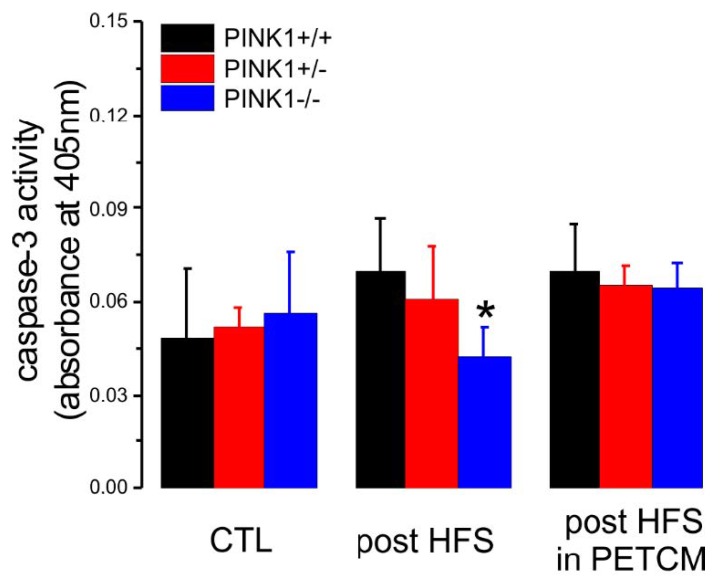
Caspase-3 biochemical assay showed low levels of caspase-3 in PINK1^−/−^ mice after HFS. Non-treated (CTL) brain slices obtained from the three genotypes did not show significant differences in caspase-3 activity levels (PINK1^+/+^: absorbance = 0.048 ± 0.023, *n* = 6; black column; PINK1^+/−^: absorbance = 0.052 ± 0.007, *n* = 3; red column; PINK1^−/−^: absorbance = 0.057 ± 0.020, *n* = 6; blue column; one-way ANOVA and Tukey post hoc test, *p* > 0.05). Caspase-3 activity measured post-HFS was significantly reduced in PINK1^−/−^ slices compared with other genotypes (PINK1^+/+^: absorbance = 0.070 ± 0.017, *n* = 6; black column; PINK1^+/−^: absorbance = 0.061 ± 0.017, *n* = 3; red column; PINK1^−/−^: absorbance = 0.042 ± 0.010, *n* = 5; blue column; one-way ANOVA and Tukey post hoc test, * *p* < 0.05). If slices were pretreated with the activator of caspase-3, PETCM, the same HFS protocol induced similar levels of caspase-3 activity (PINK1^+/+^: absorbance = 0.070 ± 0.015, *n* = 6; black column; PINK1^+/−^: absorbance = 0.066 ± 0.006, *n* = 3; red column; PINK1^−/−^: absorbance = 0.064 ± 0.008, *n* = 6; blue column; one-way ANOVA and Tukey post hoc test, *p* > 0.05). Data are represented as mean ± SD.

Next, we repeated the colorimetric assay in striatal slices obtained from the three genotypes in different experimental conditions: non-treated slices (CTRL), post-HFS slices, and post-HFS slices preincubated with low doses of PETCM (30 µM, 1 h, 32 °C) (Figure 5). We obtained similar low levels of caspase-3 activation in the CTL condition in all genotypes. Interestingly, after HFS protocol, we observed a significant decrease of caspase-3 activation only in PINK1^−/−^ mice, compared with the other genotypes. In light of these results, we treated PINK1^−/−^ slices with PETCM (30 µM, 1 h, 32 °C) before HFS stimulation. In this condition, we observed rescue of PINK1^−/−^ post-HFS caspase-3 activity to levels similar to PINK1^+/+^ and PINK1^+/−^ mice.

### 2.5. Analysis of Glutamate Vesicular Release in the Different PINK1 Genotypes

**Figure 6:**
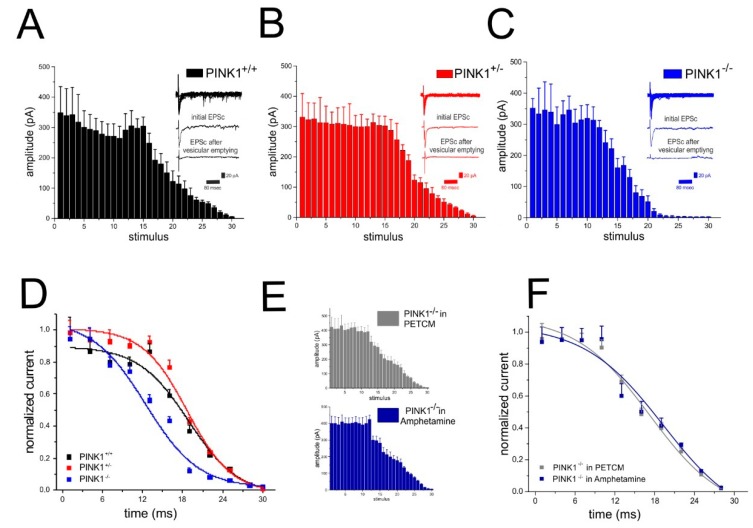
Effect of caspase-3 activation on synaptic responses to sustained electrical stimulation in PINK1^−/−^ MSNs. (**A**) Time-course of synaptic responses to sustained stimulation (30 Hz, 30 s) in PINK1^+/+^ MSNs. Current peak amplitudes are reported as mean ± SEM. In the inset, the first and last EPSC from a representative experiment are shown. (**B**) Time course of synaptic responses in PINK1^+/−^ MSNs shows a similar response to sustained stimulation. (**C**) In PINK^−/−^ MSNs, we observed a faster depression kinetics after the 12th stimulus. (**D**) In PINK1^+/+^ MSNs, the normalized current curve shows an initial fast depression, followed by a slow fall of response. The fit was used to measure the time constant of synaptic depression, which appeared slightly different in PINK1^−/−^ mice compared with PINK1^+/+^ and PINK1^+/−^ mice. Decay time constants: PINK1^+/+^: 18.88 ± 0.8 ms, *n* = 4; PINK1^+/−^: 18.68 ± 0.56 ms, *n* = 5; PINK1^−/−^: 12,43 ± 2.43 ms, *n* = 6; ANOVA and Tukey post hoc, *p* < 0.05). (**E**) Time-course of synaptic responses to sustained stimulation in the presence of amphetamine (100 µM) (blue) and PETCM (30 µM) (gray). No significant differences were observed among the three genotypes when PINK1^−/−^ was treated by either amphetamine. (**F**) No differences were detected between normalized curves obtained from PINK1^−/−^ MSNs treated with either amphetamine or PETCM. Time constants: PINK1^−/−^ plus amphetamine: 19.28 ± 0.93 ms, *n* = 6; *p* > 0.05 vs. PINK1^−/−^; PINK1^−/−^ plus PETCM: 17.22 ± 0.86 ms, *n* = 4; *p* > 0.05 vs. PINK1^−/−^.

In different animal models of monogenic PD, such as LRRK2, α-synuclein, Parkin, and DJ-1, an impairment of membrane trafficking and synaptic release has been shown [22,28,29,30,31]. This altered neurotransmitter release has been interpreted as a dysregulation of synaptic vesicle mobilization and/or trafficking. In physiological conditions, the exocytosis process ensures the correct synaptic activity, while in these forms of monogenic PD, a reduced vesicle mobilization or trafficking impairs transmitter release, thereby altering synaptic function [23,24]. To this regard, we performed additional experiments to evaluate whether PINK1 knockout could influence vesicular release. To this aim, a 30 s 30 Hz stimulation protocol [32] in PTX (50 µM) was applied to trigger multiple EPSCs. As expected, this protocol induced a synaptic depression in all PINK1 genotypes (Figure 6A–C). However, in PINK1^−/−^ slices, the decline of EPSCs amplitude showed a slightly different profile, compared with PINK1^+/+^ and PINK1^+/−^ (Figure 6D). This different synaptic depression rate can be explained by a decrease in vesicular release probability [32,33]. 

We previously demonstrated a consistent decrease in evoked dopamine release in PINK1^−/−^ striatal slices by amperometric recordings [22,23]. This release impairment was rescued by the exogenous application of amphetamine. Recently, Avelar and colleagues demonstrated that amphetamine can increase vesicular release in the dorsal striatum, by stimulating dopamine synthesis and inhibiting dopamine degradation [34]. Moreover, amphetamine induces adaptations in glutamatergic signalling, regulating the glutamate release via glutamate transporters [35,36,37]. Therefore, we tested whether amphetamine could restore neurotransmitter release in PINK1^−/−^ striatum. As expected, in 100 µM amphetamine, PINK1^−/−^ MSNs showed a vesicular release profile similar to the other genotypes, with comparable time constants (Figure 6E,F). Given the role of caspase-3 in neuronal synaptic processes, we repeated the experimental recordings in the presence of the caspase-3 activator PETCM (30 µM, 1 h). Surprisingly, in PINK1^−/−^ MSNs, PETCM treatment was able to rescue vesicular release, as witnessed by the change in time constant (Figure 6E,F). These results suggest a possible involvement of caspase-3 in striatal synaptic release.

## 3. Discussion

Several studies support the role of caspase-3 in non-apoptotic cell-functions, including synaptic structural remodeling and synaptic plasticity mechanisms. Here, we provide evidence that caspase-3 plays a modulatory role in the expression of LTD at corticostriatal synapses, both in physiological and pathological conditions. We demonstrate that in PINK1 wild type mice, which express a physiological corticostriatal LTD [22], inhibition of caspase-3 by Z-Devd-fmk prevents the induction of this form of plasticity. Moreover, the impairment of striatal LTD in PINK1 knockout mice [22] can be restored by activation of caspase-3 mediated by α-(Trichloromethyl)-4-pyridineethanol (PETCM). We also demonstrate that caspase-3 modulators per se were not able to induce any change in intrinsic excitability properties in MSNs, as well as in paired pulse modulation. A number of studies support an additional role of caspase-3, because, besides its well-known involvement in apoptotic mechanisms, it acts as a key modulator of many other neuronal processes, including regulation of synaptic plasticity [9,10]. In this respect, Li and colleagues explored the function of caspase-3 in synaptic plasticity in hippocampal neurons in a caspase-3 knockout mouse model, demonstrating that this protease, activated via the mitochondrial pathway, is necessary for LTD expression, acting through regulation of the inducible internalization of AMPA receptors [10]. Although the exact mechanism by which caspase-3 promotes synaptic processes remains to be elucidated, it has been hypothesized that a fine modulation of caspase-3 activity exists at various levels, including the synaptic terminal, where it ensures physiological cellular activities unrelated to cell death. Thus, whether the cell is directed towards apoptosis or plasticity depends on the intensity and duration of caspase-3 activation [15]. Accordingly, the signaling mechanisms involved in LTD are closely linked to those implicated in cell death and survival, and it is likely that caspase-3 acts on substrates that are involved in synaptic plasticity, as has been shown for the protein kinase Akt1 [10]. In fact, proteolysis of Akt1 by caspase-3 is not only required for cell death program, but may also represent a key molecular step for LTD induction [38].

A growing body of evidence suggests the importance of the mitochondrial apoptotic pathway in the induction of LTD [1,4,9,38]. The mitochondrial intrinsic apoptotic pathway is engaged by BAD activation, a pro-apoptotic protein, which belongs to the Bcl-2 family [39]. BAD activates BAX, which induces the mitochondrial outer membrane permeabilization (MOMP) [40], which, in turn, promotes the cytosolic release of cytochrome c, with consequent activation of caspase-9 and caspase-3. During the expression of LTD, the BAD-induced caspase activation is necessary to promote AMPA receptor endocytosis and the expression of long-term synaptic plasticity [39]. This is confirmed by experiments on BAD knockout mice, which show deficient caspase-3 activation and AMPA receptor internalization, and the absence of LTD [39,41]. BCL-XL, another member of the Bcl-2 family located on the outer mitochondrial membrane, plays an anti-apoptotic role by inhibiting BAX [38]; it has been demonstrated that also overexpression of BCL-XL inhibits LTD [10]. In this scenario, PINK1 protein can be considered a key actor too. PINK1 and BCL-XL colocalize at the outer mitochondrial membrane, and Arena and colleagues showed that PINK1 can protect cell against apoptosis by phosphorylating BCL-XL, preventing its pro-apoptotic cleavage [41]. The interaction between these two proteins would take place only on depolarized mitochondria, suggesting a specific role for this molecular mechanism in mitochondria physiopathology [41]. Moreover, data support a crucial function of PINK1 in mitochondria-dependent apoptosis [42]. PINK1 acts as a neuroprotective protein, protecting cells from damage-mediated mitochondrial dysfunction [43]. PINK1 mutations impair mitochondrial complex I activity, causing mitochondrial membrane depolarization and increased vulnerability to stress [44], and also affecting crucial cellular processes including neurotransmitter release from presynaptic terminals, which unavoidably impairs long-term synaptic plasticity [22,26,44]. Compelling evidence suggests a complex interaction among mitochondrial intrinsic apoptotic pathway, PINK1, and the long-term synaptic plasticity machinery, where caspase-3 may act as a sensor whose activation degree may determine either the cell fate or the expression of long-term synaptic plasticity.

We hypothesize that a fine modulation of caspase cascade exits at corticostriatal synapses, and that PINK1 deficiency, impairing mitochondrial functional integrity, may also perturb these downstream mechanisms, leading to synaptic dysfunction. 

As previously demonstrated, amphetamine acts as an indirect agonist of dopamine receptor, facilitating vesicular dopamine [45] and glutamate release in the striatum [46,47,48], which can explain its capacity to restore synaptic plasticity deficit in the PINK1 model [22]. Besides the well-known ability of amphetamine to increase dopamine transmission, it may also trigger activation of caspase-3 [49]. This evidence could in part explain the failed activation of caspase-3 in our knockout PINK1 model, given the link between the mitochondrial BCL-2/Beclin complex and the absence of PINK1. In this regard, our results suggest that the rescue of LTD exerted by amphetamine in the PINK1 homozygous mouse can be ascribed to caspase-3 activation. This hypothesis is further supported by the finding that caspase-3 inhibition by Z-Devd-fmk completely blocked LTD amphetamine-mediated rescue. Further studies are required to establish the precise mechanism of this involvement. However, we hypothesize that caspase 3 is involved in striatal synaptic plasticity processes in our transgenic PINK1 mouse model.

## 4. Material and Methods

### 4.1. Animals 

The Animal Care and Use Committees of University of Rome “Tor Vergata” and IRCCS Fondazione Santa Lucia, and the Italian Ministry of Health approved the experiments (Aut. Nr. 517/2016-PR). All the experiments were carried out in accordance to the Directive EU 2010/63 on the protection of animals used for scientific purposes, and the implementation of the directive by Italian legislature: DLS/26 04/03/2014. Mice were generated and characterized as previously reported [22]. Homozygous (PINK1^−/−^) and heterozygous (PINK1^+/−^) mice and their wild-type littermates (PINK1^+/+^) were bred at our animal house. All experiments were performed blindly. Body weight and animal welfare were monitored throughout the duration of experiments. For genotyping, DNA was isolated from mouse-tail using the Extract-N-Amp Tissue PCR Kit (Sigma-Aldrich, Milan, Italy) and processed as previously described [26].

### 4.2. Electrophysiology

Mice (2–3 months of age) were sacrificed by cervical dislocation and the brain was immediately removed from the skull and cut with a vibratome (Leica Microsystems, Buccinasco, Milan, Italy) in Krebs’ solution (126 NaCl, 2.5 KCl, 1.3 MgCl_2_, 1.2 NaH_2_PO_4_, 2.4 CaCl_2_, 10 glucose, 18 NaHCO_3_, all expressed in mM), bubbled with 95% O_2_ and 5% CO_2_, as previously described [25,26]. Sagittal corticostriatal slices (300 μm thick) were maintained in oxygenated Krebs’ solution for 60 min to recover, then transferred into a recording chamber, continuously superfused with oxygenated Krebs’ solution at 32–33 °C. Sharp-electrode recordings of striatal medium spiny neurons (MSNs) were performed in the current-clamp configuration, by using intracellular borosilicate electrodes filled with 2M KCl (30–60 MΩ). Signal acquisition and off-line analysis were performed with Axoclamp 2B amplifiers and pClamp 10.2 (Axon instruments, Molecular Devices, LLC. San Jose, CA, USA). A bipolar electrode was placed in the corpus callosum to evoke glutamatergic corticostriatal excitatory postsynaptic potentials (EPSPs), in the presence of picrotoxin (PTX, 50 μM), a gamma-aminobutyric acid-A (GABA-A) receptors antagonist. The current–voltage (I–V) relationship was assessed by applying 10 mV steps (0.3 s duration) ranging from −140 to −40 mV. A negative step of −10 mV was utilized to measure membrane resistance. Paired-pulse facilitation (PPF) was evaluated by delivering two stimuli at 50 ms interstimulus interval (ISI), in PTX (50 μM) and measuring the EPSP2/EPSP1 ratio (PPR). For vesicular release experiments, multiple EPSPs were evoked by electric stimulation (30 Hz stimulation frequency, 30 s duration) to induce glutamate release [50,51]. High-frequency supra-threshold stimulation (HFS, three trains 100 Hz, 3 s, 20 s apart) was delivered to induce long-term depression (LTD). For each cell, the amplitude of EPSPs was averaged and plotted over-time as percentage of the mean amplitude of pre-HFS control EPSP. A further set of recordings was performed after preincubation of corticostriatal slices with caspase-3 modulators, the inhibitor Z-Devd-fmk (5 µM) and the activator PETCM (30 µM), respectively, applied for 1 h, at room temperature [10].

Synaptic responses were also recorded in the whole-cell configuration of the patch-clamp technique. Neurons were visualized using infrared differential interference contrast (IR-DIC) video microscopy in the dorsal striatum, as described [52]. Recordings were made with an Axopatch 200 amplifier (Axon instruments, Molecular Devices, LLC. San Jose, CA, USA), using borosilicate glass pipettes (1.5 mm outer diameter, 0.86 inner diameter) pulled on a P-97 Puller (Sutter Instruments, Novato, CA 94949, USA). Pipette resistances ranged from 2.5 to 5 MΩ. Membrane currents were continuously monitored, and access resistance measured in voltage-clamp was in the range of 5–30 MΩ prior to electronic compensation (60%–80% routinely used). Data were acquired with Clampex 10.6 software ((Axon instruments- Molecular Devices, LLC. San Jose, CA, USA). The pipette internal solution included (in mM): K+-gluconate (125), NaCl (10), CaCl_2_ (1.0), MgCl_2_ (2.0), 1,2-bis (2-aminophenoxy) ethane-N,N,N,N-tetra-acetic acid (BAPTA) (1), Hepes (10), guanosine 5′-triphosphate (GTP) (0.3) Mg- adenosine triphosphate (ATP) (2.0); pH adjusted to 7.3 with potassium hydroxide KOH. Pirotoxin (50 μM) was added to block GABA-A currents.

### 4.3. Caspase-3 Biochemical Assay

Corticostriatal slices (300 μm thick) from PINK1^+/+^, PINK1^+/−^, and PINK1^−/−^ mice were weighed and homogenized with a motor-driven pestle for two twenty-strokes cycles, with freezing and thawing of samples. After centrifugation at 10,000× *g* for 10 min at 4 °C, the supernatant was assessed by Bradford assay. A total of 150 μg total proteins for each genotype was processed according to manufacturer’s protocol (Abcam AB39401). The 96-well plate was incubated at 37 °C for 60 min and the output was measured (OD 405 nm) on a microplate reader, Thermo Fisher Scientific Multiskan GO.4.3.

### 4.4. Statistical Analysis 

For electrophysiological experiments, we recorded one single MSN for each brain slice, and at least six MSNs for each experimental condition. The number of animals used was at least three for each condition. Power analysis was used to determine the sample size required for different sets of experiments, in accordance to the principle of scientific reduction (Russell and Burch 1959). All data are presented as mean ± standard error mean (SEM). Data were analyzed off-line using Clampfit 10.7 and Adalta Origin 2016. Student’s *t*-test was used to evaluate statistical differences between two groups. One-way analysis of variance (ANOVA) followed by post-hoc Tukey was used to compare groups. Two-way ANOVA was used to analyze the effect of multiple variables on all genotypes. An analysis of variance with Tukey’s post-hoc test was performed among groups. Alpha was set at 0.05; the null hypothesis was rejected for *p* ≤ alpha and the experimental hypothesis was accepted. Vesicular release was calculated using the cumulative amplitude analysis [52,53] after protocol induction of 30 Hz/30 sec [33]. The time course of synaptic responses to sustained stimulation was plotted in function of cumulative amplitude. Data plotted represent the means of 4/6 independent recordings ± SEM. Data plotted were fitted in function of time, and the time constant, relative to rapid synaptic depression, was calculated by Boltzmann fit. All differences by genotypes were analyzed by ANOVA followed by post hoc test. 

For biochemical experiments, we obtained eight different slices from each animal. Data were analyzed using Adalta Origin 2016 software. One-way ANOVA followed by post-hoc Tukey was used to compare groups. *t*-test was used for all other conditions. Statistical significant was fixed at *p* < 0.05. All the data are shown as mean ± standard deviation (SD).

### 4.5. Drug Sources

Picrotoxin, (+)−MK801, and CNQX were purchased from Tocris Cookson, Bristol, UK. The genotyping kit was provided by Sigma Aldrich, Milan, Italy. Caspase inhibitors and activators were purchased from Tocris Cookson, Bristol, UK. All other drugs were purchased from Panreac Química, Castellar del Vallès (Barcelona, Spain). Caspase-3 Assay Kit (Colorimetric) was provided by Abcam, Cambridge, UK.

## 5. Conclusions

Our findings support the idea that the mitochondrial-dependent caspase pathway plays a critical role in synaptic depression and that activity of caspase-3 is crucial for the expression of LTD in the PINK1 mouse model of PD. Indeed, in our experiments, a low level of caspase-3, completely insufficient to drive the cells toward apoptosis, could contribute to activating the molecular mechanisms involved in synaptic plasticity and could restore the impairment of LTD in our animal model. Exploring the non-apoptotic activation and modulation of caspase-3 may contribute to a better comprehension of caspases’ anti-apoptotic involvement at corticostriatal level, as well as help to clarify the mechanisms involved in synaptic failure in the pathophysiology of PD [54,55], also in view of rationale therapeutic approaches for counteracting PD progression. Furthermore, in a broader view, low-non-lethal levels of caspase-3 may be involved in cellular mechanisms relevant also for other neurodegenerative diseases [56]. The evidence of a biphasic response of caspase-3 activation gives us a new prospective to investigate the pathophysiology of neurodegenerative disorders.

## Figures and Tables

**Table 1:** Electrophysiological properties of PINK1 MSNs, in the presence of caspase-3 modulators.

	PINK1^+/+^			PINK1^+/−^			PINK1^−/−^		
	Saline	Z-Dedvd-fmk	PETCM	Saline	Z-Dedvd-fmk	PETCM	Saline	Z-Dedvd-fmk	PETCM
RMP (mV)	−85.2 ± 3.8	−90.5 ± 1.6	−89.5 ± 1.3	−87.2 ± 3.6	−88.5 ±1.6	−89.3 ± 1.7	−79.4 ± 4.0	−87.10 ± 3.1	−87.4 ± 2.2
AP amplitude (mV)	77.0 ± 3.8	75.8 ± 1.88	76.8 ± 3.03	78.4 ± 3.6	75.0 ± 2.1	73.1 ± 2.9	79.4 ± 4.0	69.5 ± 4.3	75.3 ± 2.5
Membrane resistance (MΩ)	35.2 ± 2.4	36.8 ± 1.7	38.2 ± 1.8	38.0 ± 2.0	38.9 ± 0.7	38.7 ± 1.3	35.2 ± 3.3	34.1 ± 1.2	35.6 ± 1.1
Delay to first spike (ms)	40.5 ± 1.99	41.2 ± 1.3	42.3 ± 1.6	42.1 ± 1.6	43.8 ± 1.6	42.9 ± 1.5	44.2 ± 2.0	44.7 ± 1.8	46.0 ± 0.3
Rheobase (pA)	421 ± 12	418 ± 9	444 ± 11	400 ± 18	373 ± 6	387 ± 12	380 ± 18	376 ± 6	366 ± 15

Corticostriatal slices from PINK1^+/+^, PINK1^+/−^, and PINK1^−/−^ mice were randomly assigned to saline solution (vehicle), or to saline solution plus either the caspase-3 inhibitor Z-Devd-fmk (5 µM) or the caspase-3 activator PETCM (30 µM). After 1 h pretreatment at room temperature, each brain slice was transferred into the recording chamber and continuously perfused with the same drug, during the entire experimental period. Pharmacological treatment with vehicle, Z-Dved-fmk, or PETCM did not alter MSNs’ intrinsic excitability properties, as well as the RMP (Table 1; Figure 1A–F; one-way analysis of variance (ANOVA) *p* > 0.05).

## Supplementary Materials

Supplementary materials can be found at https://www.mdpi.com/1422-0067/20/14/3407/s1.
