# Supplementary material for: Loss of Non-Apoptotic Role of Caspase-3 in the PINK1 Mouse Model of Parkinson’s Disease
Source: Int J Mol Sci. 2019 Jul 11;20(14):3407. doi: 10.3390/ijms20143407 (PMC6678522; doi:10.3390/ijms20143407)
Supplement: Supplementary file 1 [file ijms-20-03407-s001.pdf]

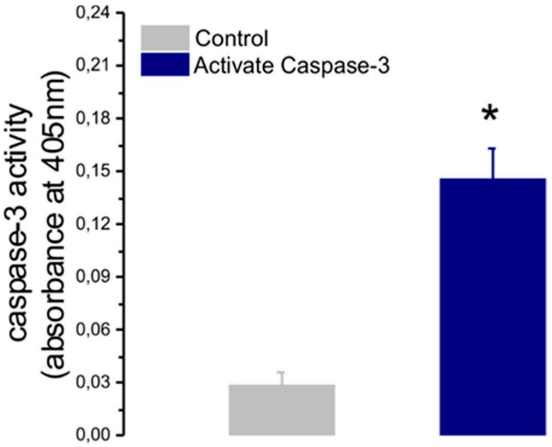

Figure S1. Caspase-3 activation was measured using colorimetric kit. Wild type slices were incubated with high doses of PETCM (100 μM, for 1h, 37 °C; navy column), and compared them with naïve samples (untreated slices; grey column). PETCM was able to induce a drastic increase in caspase-3 activity (naïve absorbance = 0.0285 ± 0.0077, n = 3; PETCM absorbance = 0.14550 ± 0.01768; n = 3; $p < 0.05$ T-test).
